# Understanding sexual healthcare seeking behaviour: why a broader research perspective is needed

**DOI:** 10.1186/s12913-017-2420-z

**Published:** 2017-07-06

**Authors:** Fiona Mapp, Kaye Wellings, Ford Hickson, Catherine H. Mercer

**Affiliations:** 1 Department of Social and Environmental Health Research, Faculty of Public Health and Policy, London School of Hygiene & Tropical Medicine, 15-17 Tavistock Place, London, WC1H 9SH UK; 2 Research Department of Infection and Population Health, University College London, Mortimer Market Centre, London, WC1E 6JB UK

**Keywords:** Non-attendance, Sexually transmitted infections, Sexual health clinics, Non-patient samples, Natsal-3, Irving Zola

## Abstract

**Background:**

Despite effective and accessible treatments, many sexually transmitted infections (STIs) in high-income countries go untreated, causing poor sexual health for individuals and their partners. Research into STI care has tended to focus on biomedical aspects of infections using patient samples and prioritised attendance at healthcare services. This approach overlooks the broader social context of STIs and healthcare-seeking behaviours, which are important to better understand the issue of untreated infections.

**Main body:**

This paper is structured around three main arguments to improve understanding of help-seeking behaviour for STIs in order to help reduce the burden of untreated STIs for both individuals and public health. Firstly, biomedical perspectives must be combined with sociological approaches to align individual priorities with clinical insights. More research attention on understanding the subjective experiences of STI symptoms and links to healthcare-seeking behaviour is also needed. Secondly, a focus on non-attendance at healthcare services is required to address the patient-centric focus of STI research and to understand the reasons why individuals do not seek care. Finally, research using non-patient samples recruited from outside medical contexts is vital to accurately reflect the range of behaviours, beliefs and health issues within the population to ensure appropriate and effective service provision. We suggest piggy-backing other research on to existing studies as an effective way to recruit participants not defined by their patient status, and use a study recruiting a purposive non-patient sample from an existing dataset – Britain’s third National Survey of Sexual Attitudes and Lifestyles (Natsal-3) as an illustrative example.

**Conclusion:**

STIs are common but treatable, however a range of social and cultural factors prevent access to healthcare services and contribute to the burden of untreated infection. Different conceptual and empirical approaches are needed to better understand care-seeking behaviour and reduce the gap between social and biomedical advancements in managing untreated infection.

## Background

Sexually transmitted infections (STIs), acquired through genital contact, have deleterious effects on an individual’s sexual and reproductive health and well-being and present a persistent public health problem. Prevention efforts are often thwarted by the complexity of factors involved in changing sexual behaviour [1], the stigmatization of both having STIs [2] and using sexual healthcare services [3] and moral opposition to teaching sex and relationships education in schools [4]. There remains a lack of evidence about the effectiveness of interventions to reduce STI prevalence, and cost effectiveness of STI prevention is a barrier to political support [5]. The World Health Organization estimates that every day, more than 1 million people acquire a STI [6]. It is difficult to quantify the proportion of these infections which go undiagnosed and untreated, which is part of the problem of understanding and managing untreated infections. Empirical studies suggest that less than 50% of untreated chlamydia spontaneously resolves without treatment [7, 8] while several viral STIs including HIV, herpes, hepatitis B and human papillomavirus are incurable. Modelling studies have estimated that if sex partners are not treated simultaneously, 19.4% of patients diagnosed with chlamydia and 12.5% of those with gonorrhoea will be re-infected [9]. Untreated infections can cause long-term health problems exacerbating the burden of STIs globally. Herpes and syphilis can increase the risk of acquiring HIV by three-fold, chlamydia and gonorrhoea have been linked to pelvic inflammatory disease which in turn can result in infertility, particularly if symptomatic [10] and human papillomavirus causes 528,000 cases of cervical cancer annually [6, 11]. Chronic pelvic pain, ovarian abscesses and ectopic pregnancies are not uncommon sequelae [12]. Mother-to-child transmission of STIs such as syphilis can result in stillbirth, neonatal death, prematurity, congenital deformities and neonatal infections including pneumonia and conjunctivitis [6]. STI surveillance in high-income countries is well established and national monitoring agencies regularly report infection prevalence, trends over time, clinic attendances [13] and undiagnosed HIV [14] (estimated using models based on population surveillance data e.g. [15] and surveys of children, pregnant women and high risk groups [16]). STIs have considerable economic impact on healthcare systems with recent estimates suggesting an annual cost of almost $16 billion in the USA [17]. Projected costs of STIs excluding HIV in the UK between 2013 and 2020 are more than £6 billion ($7.7 billion) based on access levels at the time [18], however severe funding cuts have led to reduced service accessibility [19]. Given that the potential physical, economic and emotional burden of STIs is considerable and that effective treatments for the majority of infections exist and are free at the point of access in high-income countries, an important question remains unanswered: how does individual sexual healthcare-seeking behaviour contribute to untreated STIs?

Asymptomatic infection is a major contributory factor to untreated infections. One study of 18–29 year olds in Louisiana estimated that 45% of all gonorrhoea and 77% of all chlamydia cases were never symptomatic and lack of symptoms was the most important reason for infections going untreated [20]. Similar results have been reported in other populations (e.g. [21]). These findings emphasise the importance of STI screening programmes as part of national STI management strategies. However, the problem of untreated STIs goes beyond infections which do not produce symptoms, and other aspects of STIs must be addressed to help reduce prevalence and improve sexual health and wellbeing. Mercer et al. found symptomatic individuals attending specialist sexual health clinics in England were significantly more likely to have an acute STI diagnosed than those not reporting any symptoms [22], highlighting the need to better understand experiences of STIs. Further discussion of asymptomatic STIs is beyond the scope of this debate.

In the UK, STIs can be diagnosed and treated in specialist sexual health clinics (or genito-urinary medicine (GUM) clinics), primary care (General Practice surgeries), antenatal services, and other community settings [23–25]. Service provision has been broadened with initiatives such as the National Chlamydia Screening Programme [26] and the development of self-sampling and self-testing kits for use at home, such as the SH:24 project [27]. Accessing these services requires individuals to seek care or at least engage with opportunistic STI testing. Here we focus on seeking care in response to STI associated symptoms. We start by arguing that STI research must expand beyond biomedically dominated perspectives of infections to encompass socially oriented experiential aspects of genito-urinary health and care-seeking, using symptoms as a key example. This informs our second line of argument that focussing on non-attendance as part of sexual healthcare-seeking is important to understand why some people do not use available services. Finally we suggest that sampling strategies for STI research should be inclusive of people who do not access health services. We review different sampling approaches that facilitate non-patient sample recruitment and discuss ‘piggy-backing’ research on to existing studies as an under-used option to recruit participants independently of healthcare settings.

## Combining biomedical and sociological lenses to prioritise symptom experiences in research

Research on genito-urinary conditions tends to be biomedically framed with a focus on developing effective treatment regimens and identifying risk factors to help target health promotion initiatives [5, 11, 21]. Although essential to preventing STIs, biomedical perspectives overlook the meaning and significance of symptoms and diagnoses to individuals, the sense-making processes that take place in response to these experiences and the implications of being treated for a stigmatising condition. The biomedical dominance in this area of health research calls for balance with other perspectives to progress understanding about genito-urinary health issues and link understanding about diseases, people and health care services.

Medical sociology has reframed health and illness research topics to focus on the experiential, social and lived realities adding a variety of lenses through which to examine phenomena [28]. The intense research efforts around HIV have shown the disease has both social and biomedical significance [29] but the two perspectives have not been well-integrated. The biomedicalisation of HIV has neglected the social significance of prevention, treatment and care [29] and this trend has spread into STIs. The gap between knowledge gained and direct impact on health of individuals and populations will continue to expand unless social research on genito-urinary conditions is considered in conjunction with biomedicine. There is a need to unite the physical and social body in the context of healthcare-seeking, in line with recent calls for more emphasis on the person with the medical problem, not the clinical problem itself [30].

Irving Zola started linking symptom experiences with what individuals did about them by rejecting common assumptions pertaining to health issues and service use [31]. His three explanations of the relationship between symptoms and care-seeking are well known and have been widely applied to health and social research. Firstly, Zola suggested that people will have a symptom(s) of something the majority of the time but most are considered too minor to warrant medical treatment; secondly, that the seriousness and frequency of symptoms do not predict attendance (highlighting the discrepancy between medical and social interpretations of bodily experiences); and thirdly, that most people make rational decisions about seeking, delaying or not attending care based on their own belief system and internal values (ibid). Zola was interested in the point at which symptoms could no longer be tolerated physically, personally and socially resulting in help-seeking behaviour. His work emphasises the importance of experiential aspects of conditions and he noted that *“the ‘illness’ for which one seeks help may only in part be a physical relief from symptoms”* ([17] p.679). This suggests that the need for a combined approach to understand the underlying pathogen as well as the social implications of the disease are well established but poorly implemented in relation to sexual health.

Experiences of disease and illness are broader than those observed in a clinical environment. The lived experience of a health condition is not the same for everyone affected by it and experiences may contradict population patterns and statistical associations, especially of risk factors. There have already been calls for an increased focus on personal experiences and data on the lived realities of health conditions [32]. Additionally, proposed changes to the International Classification of Diseases (ICD-11) emphasise the importance of subjective experiences of patients, embodying a more integrated approach to sexual health practice [33], which research should reflect. This turn towards individualising health experiences helps capture the diversity of health and illness instead of homogenising the population into a series of risk-factors or sub-groups based on behaviour or other attributes. Adding a sociological lens to biomedical insights about STIs helps expand perspectives and foreground key social factors including lay explanatory frameworks of infections and symptoms, and situated rationalities in healthcare decision-making. Combining perspectives helps align the priorities of patients and providers to better address STI management.

Symptoms associated with some STIs are a key aspect of the lived experience but are frequently overlooked in STI research because of the high prevalence of asymptomatic infection [22, 20] and focus on other factors such as sexual risk behaviour [21]. Symptoms can trigger help-seeking [34, 35] and have been reported as the most common reason for attendance at healthcare [36]. Symptoms are still routinely used to triage patients in sexual health clinics and form part of a sexual history taken by healthcare professionals during clinical consultations [37]. Certain symptoms can disrupt ideas of the self, causing feelings of self-disgust, loss of innocence and shame [34, 38]. Disruption and discomfort in day-to-day life form part of the burden of STIs as well as the more long-term harmful consequences. Symptoms, such as ulcers, painful or frequent urination and itching are often not recognised as associated with STIs and mis-attributed to other causes such as yeast infections or trauma [35, 39]. This trend is also reflected for gynaecological cancer [40] suggesting overlapping symptomatology combined with lack of understanding about how genital sensations and symptoms are made sense of may represent additional barriers to seeking care and receiving treatment. There is a need to improve understanding of symptoms and identify which parts of these experiences are important to individuals and their preferences for clinical intervention (if any).

## A focus on non-attendance

Given that clinical evidence alone is insufficient to improve sexual health and individual preferences for healthcare are becoming a priority [30], we also need to consider those who do not attend services. This is not a common approach, as health research tends to use patient populations and report the experiences of those who attend services. Zola’s work [31] exemplifies the patient-centric focus of studying help-seeking and treatment issues. Non-attendance or decisions to abstain from medical care are less well researched but important in the context of untreated infections. Does non-attendance equate to an absence of help-seeking or are choices about sexual healthcare bound up with other social, psychological, cultural and biomedical factors that warrant further investigation, as Zola hinted at but did not fully explore [31]?

An alternative approach in healthcare research is asking participants hypothetical questions about their behaviour such as that posed in Britain’s third National Survey of Sexual Attitudes and Lifestyles (Natsal-3): *“If you thought that you might have an infection that is transmitted by sex, where would you first go to seek diagnosis and/or treatment?”* ([20] p.77). This question highlights the inherent expectation of a link between infection uncertainty and seeking diagnosis and/or treatment. Here we see the research process itself reinforcing the privileging of professional perspectives. There is a wealth of literature evidencing the discrepancy between aspirations or intentions to act and actual behaviour or action [41] and requires participants to project themselves into a different situation and imagine how they would respond, which may be influenced by social desirability bias. Aspirational or hypothetical questions have only limited potential for understanding the individual and social processes that take place to make sense of experiences and seek healthcare.

Care-seeking is a well-documented, complex social process involving symptom perception, interpretation, appraisal and decision-making linked to the ability and motivation to access healthcare [42]. The end-point of care-seeking is often viewed in terms of attendance at healthcare services or resolution of a clinical issue [43]. STI stigma is implicated in sexual healthcare-seeking and has been cited as the most significant barrier to accessing sexual health services [44]. Clearly not everyone with a need for care reaches a service [42] which is a major aspect of untreated STIs, but the social processes of not attending, or avoiding seeking care, have been overlooked in favour of reporting outcomes of care-seeking behaviour. In part, the lack of critical examination of not seeking care results from an over-reliance on service-user populations in health services research and the difficulty of identifying individuals who have a need for care but who have not attended services. This mis-represents the population in need of care by privileging the characteristics, needs and opinions of those who have attended services over those who have not. Little progress has been made in examining symptom experiences and care-seeking responses outside of medical settings. We therefore need new approaches and different types of data to investigate stigma mechanisms and the social context of non-attendance behaviour, from participants recruited independently of healthcare services.

## Approaches to recruiting a non-patient sample

Much of the research on STIs and healthcare-seeking to date has used samples drawn from service-user and patient populations recruited through medical settings (e.g. general practice or sexual health clinics) and/or by using hospital records. The attractions of such designs are apparent. There is a ready-made sampling frame of registered patients, attendances within a given time frame or diagnoses of a specific condition. Service-user samples are usually simple to identify and recruit, especially if there is an existing collaboration between service and research teams. Services may also hold other linkable medical records, which, with appropriate consent, can be used for research. Data collection can occur in the clinical setting whilst the person is waiting for their appointment or as a follow-up after they have seen a healthcare professional. However, the setting of data collection is known to influence the type and nature of the data produced, particularly for qualitative studies [45]. Medical settings tend to be formal, structured environments with inherent power relations between patient and professional, which may extend to the researcher and result in an implicit social hierarchy. Participants are viewed first and foremost as patients as well as informants on the research topic and this necessarily influences the data produced.

Using patient samples focuses exclusively on people who attend healthcare services who are known to differ from those who do not attend care [46], impacting on the data generated. Non-patient samples are vital for understanding unmet needs as well as service use and should be used more widely than they currently are. Sampling a more diverse range of individuals with healthcare needs is necessary for public health to enable better descriptions of health issues, behaviours, attitudes and decision-making. A non-patient sample is likely to mean different things in different contexts and for different research studies, as the vast majority of people living in high-income countries have experienced some clinical interaction during their life. Research looking at selective care-seeking or avoidance of specific care settings will need to accommodate the diversity of care-seeking behaviour and use innovative and opportunistic methods to define and recruit the sample.

Non-patient samples are under-used partly because of the difficulties in defining the sampling frame. The denominator may be the whole population or it may be a sub-group with a specific characteristic but if the data are not routinely collected along with contact details, the sub-group is less visible to researchers and recruitment becomes more difficult. Non-patient samples when they are used, are often convenience samples drawn from community settings such as sports and social clubs (for example Bourne and Robson’s study [47] exploring experiences and social constructions of safe and unsafe sexual behaviour). Targeting events including Gay Pride as well as gay clubs and bars facilitated recruitment of participants for a cross-sectional annual behavioural survey of gay men [48, 49] and online recruitment is also a common approach [50]. Methodologically this enables data collection to take place away from a healthcare environment and can give useful insight into the topic but these sampling approaches are not systematic as they select the most accessible participants who often have a pre-existing interest in the topic. When considered in the context of the hierarchy of data debates in social science research [51], convenience samples lack credibility when compared to purposive, theoretical and probability samples [52].

Recent innovations in sampling techniques mitigate some of the limitations of convenience samples and balance efficient recruitment with sample representativeness. Two techniques enable non-patient samples to be recruited through approaches seeking to approximate probability sampling. Firstly respondent-driven sampling combines snowball sampling with a mathematical model that weights the sample to compensate for the non-random approach. It also introduces statistical rigor by using longer recruitment chains and by imposing recruitment limits [53, 54]. Respondent-driven sampling has been widely used for biological and behavioural studies of HIV worldwide [55] but applications of this method are limited as the population need to be socially networked with clear eligibility criteria, and equilibrium (ensuring bias is not introduced because of the snowball sampling) must be achieved [56]. Secondly, time location sampling is useful for accessing hard to reach groups by mapping locations they frequent and randomly selecting the day, time and place to systematically select participants. This method was used effectively to estimate the prevalence of STI-associated symptoms and care-seeking patterns in street-based surveys in Iran [57], taking into account cultural sensitivities and the absence of valid data about STIs. However, time location sampling is limited to people attending venues in the sampling frame during the sampling period with associated resource implications for the study [58]. Whilst a welcome addition to the toolbox for sampling different types of populations, both of the sampling methods discussed are for quantitative data collection and rely on social interaction – either through peers or at a venue. Additionally they rely on the key characteristics of interest being visible and/or easily disclosed by the population. Therefore neither approach is suitable for targeting sensitive issues in a diverse sample from the general population.

As there are limited options for sampling people who are not defined by their service-user status, there is a need for methodological innovation to create other opportunities for sampling and recruiting participants with potential unmet care needs outside of healthcare settings. One alternative approach is ‘piggy-backing’ additional research studies on to existing surveys of the general population (or other sub-samples) to add value and capitalise on data already collected. In this way, theoretical and purposive qualitative samples can be drawn using specific characteristics identified in the earlier survey. Providing the necessary ethics approval and governance protocols are put in place, it is possible to follow-up with people already recruited for a study. There is a growing trend towards data re-use and secondary analysis of existing data sets facilitated by increasing opportunities to access and link data through the UK Data Archive [59]. Secondary analysis of available datasets which include relevant variables is an efficient use of existing data and can produce additional insights and add value to the original study. Myers [60] built a theoretical framework of reasons for STI testing from existing qualitative research to inform analyses of publically available data from a nationally representative survey of adolescent health, with participants recruited from U.S. schools. The findings confirmed the importance of STI symptoms as well as concerns about recent sexual behaviour as predictors of STI testing in young women, without needing to collect more data. Similarly, van Bergen [61] used sexual health questions embedded within the second Dutch National Survey of General Practice to describe the prevalence and distribution of STIs, symptoms and healthcare-seeking behaviour in the Netherlands. Both Myers [60] and van Bergen [61] used non-patient samples to quantitatively explore issues related to STIs in nationally representative data, but explanations for these behaviours and experiences from those participants who reported them are missing in single method approaches. To assess the influence of non-help-seeking behaviour on untreated STIs, we need to understand the importance of socio-cultural factors in care-seeking, the prevalence, interpretation and management of symptoms and reasons for not seeking care.

We took a different ‘piggy-backing’ approach to expand our conceptualisations of care-seeking for STI symptoms using a sequential mixed methods study design [62]. Analyses of data from the third British National Survey of Sexual Attitudes and Lifestyles (known as Natsal-3) [63, 64] produced population estimates of the variables of interest and helped identify and recruit a sample for follow-up interviews. We therefore identified a sampling frame for follow-up explanatory qualitative research using quantitative variables to identify characteristics of interest amongst survey participants, enabling us to recruit the same participants into a second wave of data collection and produce linked datasets. Natsal-3 is a probability survey and asked questions about STI symptoms and care-seeking behaviour as well as detailed demographic and other behavioural and attitudinal questions relating to sexual health [65]. We sampled individuals who reported one or more STI symptoms and had never attended a sexual health clinic [66]. Specifically we were able to access individuals with potential care needs and explore care-seeking responses using linked survey data and semi-structured interview data to examine areas of convergence, divergence and silence [67]. We were also able to investigate men’s experience of genito-urinary symptoms which have been noticeably absent from the literature. Similar studies using linked data from national surveys have been carried out such as a qualitative study about growing up in step-families with participants selected from the British birth cohort study of 1958 [68].

Both of these study designs involved sequential data collection and were opportunistic uses of existing data to explain earlier findings and enrich our knowledge of the specific social phenomena being studied. More research opportunities may be created through collaborations with other national observational studies or existing sampling frames of specific populations. Additional studies using innovative sampling methods are likely to improve the representation of historically under-researched populations and help reassure potential participants of the importance of the research, encouraging participation. Researchers must be aware of the possibility of research fatigue in participants and disengagement from the process, or the development of ‘expert’ participants who have significant experience of taking part in research as these participant characteristics will affect the data produced. Furthermore, nesting research in existing studies can result in a time lag between different data collection phases causing high levels of participant attrition, and the sampling frame for subsequent studies is restricted to the variables included in the original dataset.

Clearly there will be specific ethical, governance and access issues to think through for using piggy-backing approaches, but the time and resource savings realised in our approach to researching people with a need for care who do not attend services, outweigh limitations. The wealth of robust data we have from national surveillance and surveys [60] could and should be capitalised on to advance our understanding of healthcare issues outside of medical settings and facilitate more sociological investigation of these phenomena. While exploring alternative sampling strategies is vital to drive progress in understanding issues pertaining to healthcare-seeking and untreated STIs, work to convince funding bodies is still needed to prioritise and invest in methodological innovation and non-patient sampling frames to facilitate empirical research.

## Conclusions

STIs are common and persistent health issues requiring multi-perspectival insights to increase diagnosis and treatment rates and help tackle current global trends. There is an urgent need to improve understanding about STI symptoms and the social and cultural factors governing care-seeking behaviour, in conjunction with biomedical knowledge of pathogens. These factors are not well understood due to the fragmentation of STI research and the lack of collaboration between biomedical and social scientists to address the gaps in knowledge. There is still an over-reliance on patient samples but opportunities to innovate and generate data outside of medical settings using participants not primarily identified by their patient status will help to understand the phenomena of untreated STIs in the context of care-seeking behaviours. Piggy-backing approaches offer promising new ways to sample participants whilst minimising additional resource implications and mixed methods studies in particular offer opportunities to gain comprehensive insights into issues around STI care using linked data.

## Abbreviations

GP: General practice/practitioner
GUM: Genito-urinary medicine
HIV: Human Immunodeficiency Virus
Natsal-3: Britain’s third National Survey of Sexual Attitudes and Lifestyles
STI: Sexually Transmitted Infection
